# A Multi-Modal Dataset for Automated Phenological Stage Mapping in *Actinidia chinensis*

**DOI:** 10.1038/s41597-026-07360-7

**Published:** 2026-05-21

**Authors:** Isabel Pinheiro, Pedro Moura, Leandro Rodrigues, Germano Moreira, Rui Manuel Coutinho, Francisco Terra, António Valente, Mário Cunha, Filipe Neves dos Santos

**Affiliations:** 1 Institute for Systems and Computer Engineering, Technology and Science (INESC TEC), Porto, 4200-465 Portugal; 2 School of Science and Technology, University of Trás-os-Montes e Alto Douro (UTAD), Vila Real, 5000-801 Portugal; 3 Faculty of Sciences, University of Porto (FCUP), Porto, 4169-007 Portugal; 4 Faculty of Engineering, University of Porto (FEUP), Porto, 4169-007 Portugal

## Abstract

Phenological monitoring of Actinidia chinensis is critical for optimising operational costs and yield prediction. However, current manual assessment methods are time-consuming, making them impractical for large-scale precision agriculture applications. Most existing phenological datasets focus exclusively on image data without spatial validation. The Multi-Modal Actinidia chinensis Phenology Dataset is composed of (i) 1 665 annotated images of phenological stages from bud to fruit set and (ii) georeferenced videos with systematic manual ground truth of spatial stage distributions. The dataset employs an adapted 17-class BBCH system that consolidates visually similar stages, excludes problematic categories, and introduces generic structural classes to address practical annotation difficulties. Additionally, the data is organised hierarchically across various plant structures, genders, and phenological stages. The annotated images offer versatility for a range of applications, including training data for computer vision models to detect phenological stages. Furthermore, the georeferenced videos facilitate the validation of automated counting algorithms. This combined approach enables plant-level detection accuracy and provides an illustrative methodology for spatial validation that users can extend to additional orchards, promoting the development and benchmarking of automated phenological monitoring systems for precision agriculture applications in kiwifruit production.

## Background & Summary

Phenological monitoring in orchards relies on labour-intensive manual assessment methods that limit comprehensive evaluation. Fruit set rate estimation and assisted pollination timing decisions require manual counting of buds, flowers and fruits across small representative areas, with each square meter requiring approximately 10 minutes of assessment time. This sampling approach introduces significant limitations, as orchards exhibit spatial heterogeneity in flowering timing and fruit development that cannot be captured through localised observations of small representative areas, since orchard-wide generalisations are imprecise and potentially suboptimal for management interventions.

Existing phenological datasets employ various monitoring approaches spanning multiple spatial and temporal scales. Image-based datasets for crop phenology have been developed for species including raspberry^1^, apple^2^, cotton^3^, and tomato^4^, utilising computer vision (CV) techniques for stage classification and yield estimation. Temporal monitoring approaches integrate unmanned aerial vehicle imagery for repeated field-scale observations^5^ and ground-based camera networks for continuous phenological tracking across diverse ecosystems^6,7^. Continental-scale vegetation monitoring has been established through satellite remote sensing^8^ and citizen science observation platforms^9^. Within *Actinidia* species, prior work has focused on flower detection and gender classification^10^.

However, these existing approaches share a standard limitation whereby datasets focus exclusively on image data without spatial validation capabilities. At the same time, satellite-based methods lack the resolution to validate patterns at the individual plant level. For *Actinidia* specifically, no dataset currently provides both detailed phenological annotations and spatially-referenced validation data. The Multi-Modal *Actinidia chinensis* Phenology Dataset^11^ is publicly available and addresses these gaps by providing georeferenced videos with manual ground truth counts, while maintaining a robust set of manually labelled images. While the image component captures morphological diversity across five orchards, the georeferenced video component demonstrates the spatial validation methodology at a single orchard and serves as an illustrative example rather than a generalizable spatial ground truth. This dual-component approach enables assessment of phenological heterogeneity at the orchard level while maintaining accuracy at the individual plant level.

The Multi-Modal *Actinidia chinensis* Phenology Dataset^11^ comprises two complementary components organised hierarchically across plant structures, gender, and phenological stages. The labelled images component provides 1 665 images with bounding box annotations for developing CV detection models. The georeferenced videos component provides spatial validation by systematically counting across defined sampling zones to benchmark automated counting algorithms. This modular implementation enables diverse applications, including phenological mapping for timing of agricultural operations such as thinning or assisted pollination, and fruit set assessment for yield prediction. Specifically for assisted pollination, phenological mapping enables the identification of receptive-flower distributions across the orchard, supporting targeted pollen-application strategies at optimal dates to maximise pollination efficiency, minimise pollen expenditure, and improve fruit quality.

**Fig. 1:**
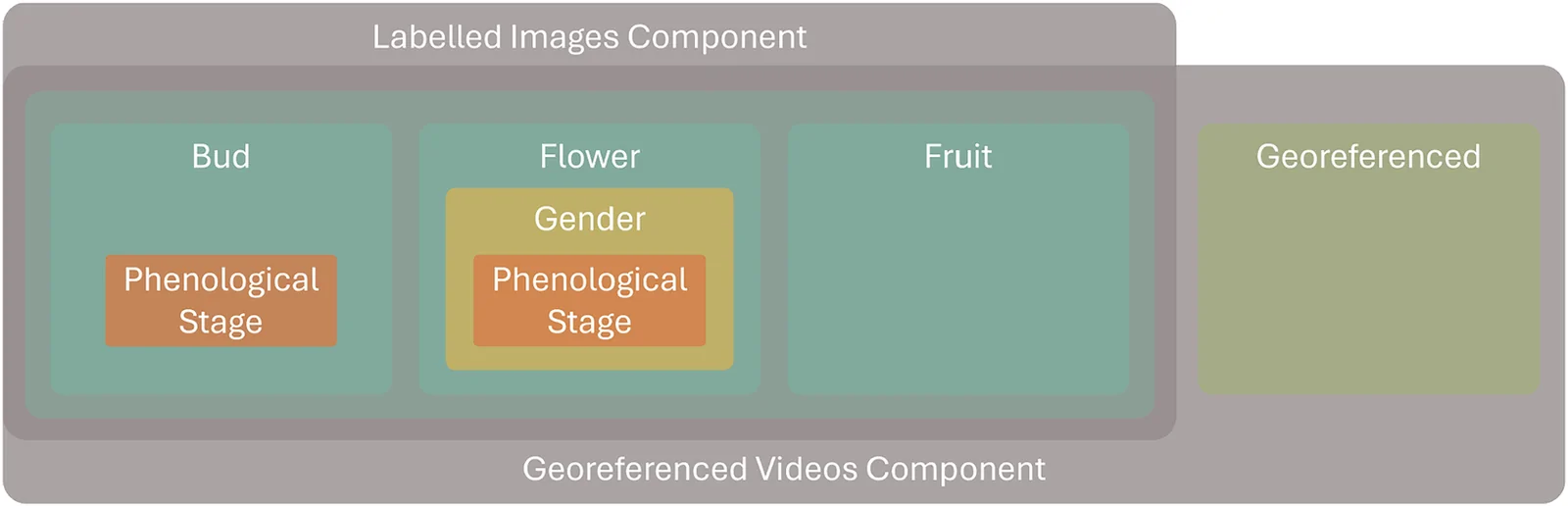
Multi-Modal *Actinidia chinensis* Phenology Dataset architecture illustrating the hierarchical organisation across two complementary components: (i) manually annotated images organised by plant structure, gender and phenological stage, and (ii) spatially referenced video recordings with systematic manual counts using the same structure.

Figure 1 illustrates the Multi-Modal *Actinidia chinensis* Phenology Dataset architecture comprising the Labelled Images Component and Georeferenced Videos Component. Both components employ a colour-coded schema to delineate the hierarchical organisation: (i) structure in blue, (ii) gender in yellow, and (iii) phenological stage in orange.

This Data Descriptor details the creation and structure of the Multi-Modal *Actinidia chinensis* Phenology Dataset in three main sections. The Methods Section presents the data-acquisition methodology, including the phenological-stage-based classification framework and the annotation protocols for both dataset components. The Data Records Section details the dataset organisation, file specifications, and repository structure. The Technical Validation Section describes the validation procedures ensuring annotation quality and reliability.

## Methods

### Data Acquisition

The data acquisition procedures were designed to address the specific requirements of the dataset’s two components described above. The strategy for labelled images prioritised morphological and environmental diversity across multiple orchards and dates to capture morphological variability. At the same time, the georeferenced videos component focused on comprehensive spatial coverage within defined sampling zones to support validation of automated monitoring algorithms.

#### Labelled Images Component

The labelled images component provides morphological annotations for developing CV models. The images were collected to maximise morphological and environmental variability in spatial, temporal, and technical parameters.

The data were collected from five different *Actinidia chinensis* orchards in northern Portugal, covering variations in age, variety, cultivar, training system, and region. Table 1 characterises the five orchards used to acquire images.

**Table 1:** Characteristics of the five *Actinidia chinensis* orchards used for data collection.

Orchard	Age (year)	Variety	Cultivar	Training system	Location (lat, long)	City
O1	12	*deliciosa*	“Hayward”	Pergola	41°19′18.14″N, 8°39′42.00″W	Vila do Conde
O2	30	*deliciosa*	“Hayward”	Pergola	41°7′35.88″N, 8°32′55.44″W	Gondomar
O3	15	*deliciosa*	“Bo.Erika”	Pergola	41°3′17.75″N, 8°35′16.99″W	Vila Nova de Gaia
O4	8	*deliciosa*	“Hayward”	T-bar	40°36′0.53″N, 8°32′40.29″W	Aveiro
O5	5	*chinensis*	“Dori”	T-bar	40°36′0.54″N, 8°32′34.61″W	Aveiro

Sampling across orchards with different ages, training systems, and regions captured morphological variability in flower arrangement and canopy structure, ensuring the dataset represents diverse production conditions. Regarding variety, the same variety does not present apparent differences in buds and flowers. However, the flowers are easily distinguished between varieties, since *Actinidia chinensis* var. *chinensis* has slightly smaller and more delicate flowers.

The images were collected over nine days on 18 May 2023 and between 17 April 2024 and 5 June 2024. This period corresponds to the flowering phase, which is the most critical for guiding agronomic practices such as assisted pollination timing and fruit thinning decisions, capturing the complete sequence of reproductive development from bud emergence to fruit set. Three operators acquired images using smartphones (iPhone Xr (SP1), iPhone 15 Pro (SP2), or Xiaomi Redmi Note 12 (SP3)) with resolutions ranging from 1080 × 1080 to 6144 × 6144 pixels. The use of multiple smartphone models introduced variability in image characteristics (sensor specifications, colour rendering, and compression), enriching the dataset by reflecting real-world acquisition conditions while maintaining sufficient image quality for reliable morphological annotation across all devices. During acquisition, operators varied the distance from the vegetative wall and the viewing angle to ensure comprehensive morphological documentation from multiple perspectives. This intentional variability captures diverse acquisition scenarios representative of practical field conditions. Temporal metadata and precise georeferencing were not recorded, as the focus was on morphological diversity rather than spatiotemporal mapping. Table 2 shows the schedule of the nine image-acquisition sessions, listing the date, orchard, operator, and smartphone model for each.

**Table 2:** Characteristics of the nine image-acquisition sessions conducted across five *Actinidia chinensis* orchards (O1-O5).

Date	Orchard	Operator	Smartphone	Images
2023-05-18	O1	OP1	SP1	576
2024-04-17	O2	OP1	SP2	237
2024-04-21	O4	OP1	SP2	252
2024-04-23	O2	OP3	SP3	29
2024-04-25	O4	OP3	SP2	139
2024-04-25	O5	OP3	SP2	574
2024-05-03	O2	OP1	SP2	238
2024-05-10	O2	OP1	SP2	394
2024-05-23	O2	OP2	SP3	193
2024-05-23	O3	OP2	SP3	209
2024-06-05	O2	OP2	SP2	263

Each session involved an operator (OP1 - OP3) with a smartphone (iPhone Xr (SP1), iPhone 15 Pro (SP2) or Xiaomi Redmi Note 12 (SP3)).

A total of 3104 images were acquired, documenting the developmental stages from bud to fruit set. This collection includes 576 images from 2023^12^ and 2528 images from 2024^13^, publicly available. From this set of images, 1665 images were selected based on uniqueness calculations using the Fiftyone platform^14^ to identify and remove duplicate or near-duplicate images, thereby avoiding accidental concept imbalance that can bias the model’s learning. The selected images were pre-processed and standardised to a resolution of 1024 × 1024 pixels to optimise compatibility with CV structures.

#### Georeferenced Videos Component

The georeferenced videos component provides spatial distribution data and manual ground truth validation for phenological mapping applications.

**Fig. 2:**
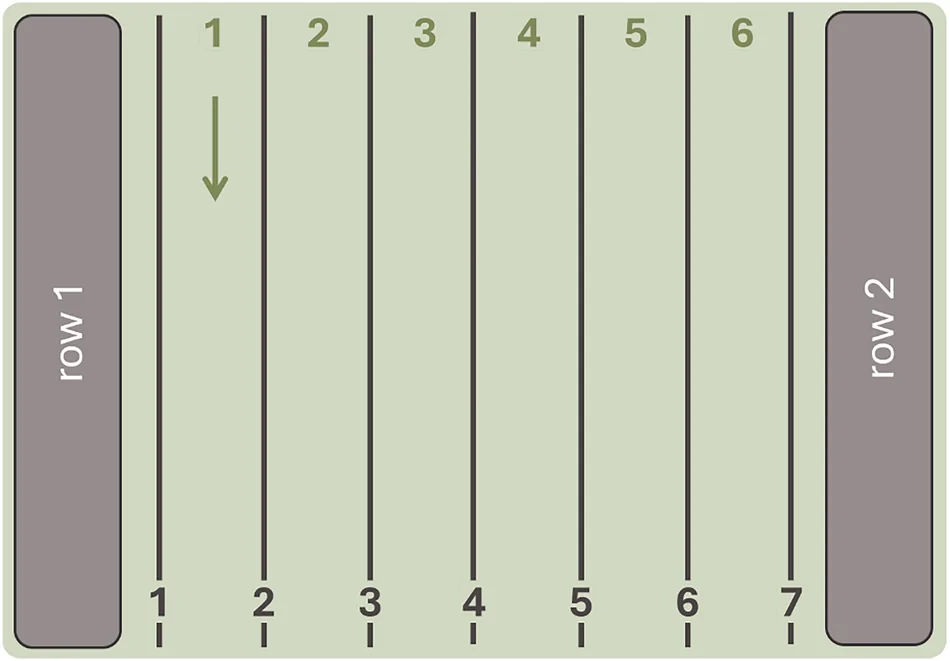
Schematic representation of the seven-wire pergola infrastructure within each sampling zone, creating six inter-wire sections that define the systematic sampling framework.

Data were collected at orchard O6, an *Actinidia chinensis* var. *chinensis* cv. “Hayward” site with 12-year-old plants trained on a pergola system, located in Vila do Conde (41°17′27.48″N, 8°38′57.57″W). Video acquisition occurred on a single date (28 May 2025) during peak reproductive activity, providing an illustrative demonstration of the spatial validation methodology rather than a comprehensive representation of spatial heterogeneity across multiple orchards or management conditions. The experimental design utilised four 5 × 5 meter sampling zones distributed across the orchard to capture within-orchard spatial heterogeneity in phenological development. Figure 2 illustrates the seven-wire pergola infrastructure within each sampling zone, creating six inter-wire sections that define the systematic sampling framework for spatial analysis.

Video acquisition was conducted on 28 May 2025 during peak reproductive activity, using standardised collection protocols. Six videos were recorded per zone along predetermined transects parallel to the vegetative canopy. The camera was mounted on a tripod positioned approximately 1.80 metres from the vegetative wall, oriented parallel to the canopy, with the operator walking at approximately 0.25 metres per second between waypoints recorded using the Geo++ RINEX Logger smartphone application^15^, with GNSS data post-processed to achieve centimetre-level accuracy. This standardised setup minimised operator-specific biases arising from height or camera-angle variability. Videos were captured using Xiaomi Poco X6 Pro (SP4) at 3840 × 2160 pixel resolution and 30 fps, stored in MP4 format. Frame-level georeferencing was achieved by linearly interpolating between the start and end coordinates, assuming constant walking velocity and a straight-line trajectory. GPS Exchange Format (GPX) files were generated for each recording, containing calculated temporal-spatial metadata for individual frames.

### Data Annotation

The Multi-Modal *Actinidia chinensis* Phenology Dataset follows a hierarchical classification framework that accommodates three levels of analysis (structure, gender, and phenological stage).

The first level distinguishes between bud, flower and fruit, three structures of *Actinidia chinensis*. The bud represents the initial stage of development, in which the floral organs are immature and not visible, and the flower develops. The flower represents the stage where the reproductive organs are visible, and individuals can be classified as female or male. When properly pollinated, the female flower develops into fruit, the final stage.

**Fig. 3:**
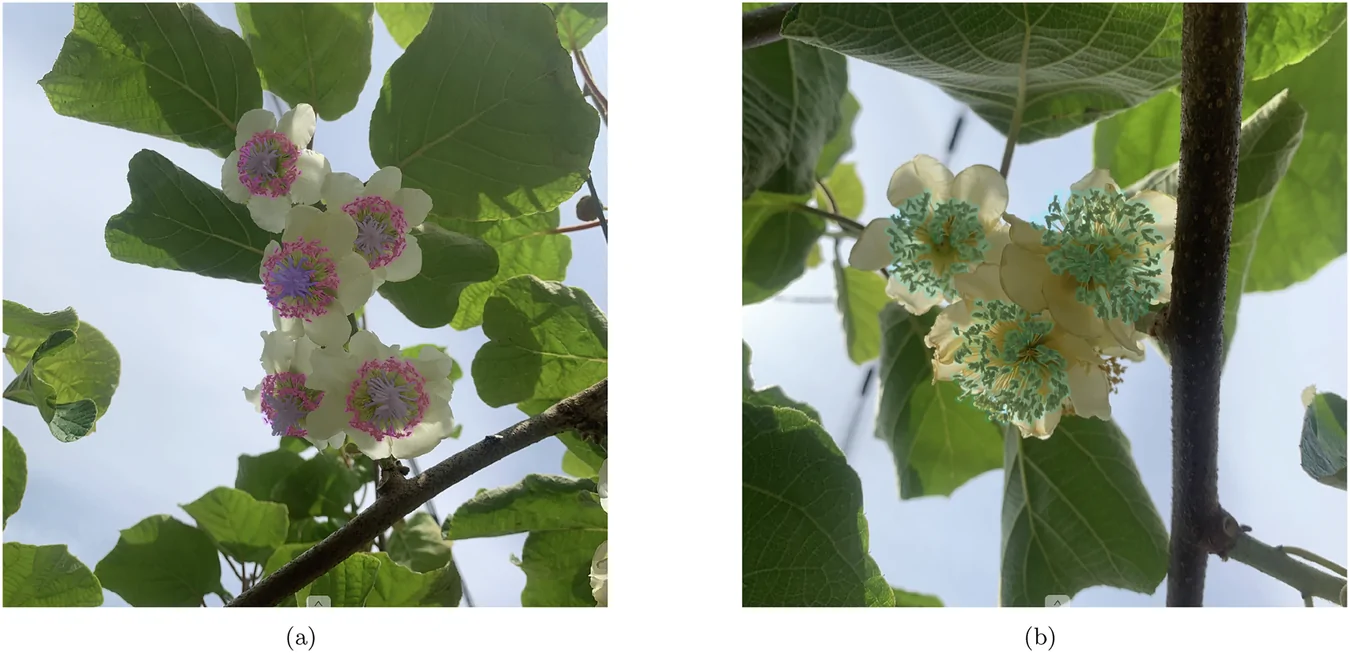
Key morphological characteristics to distinguishing gender in *Actinidia chinensis* flowers^10^. (**a**) Female flowers exhibit a star-shaped stigma (purple shadow) surrounded by the anthers that contain non-viable pollen (pink shadow). (**b**) Male flowers display numerous well-developed anthers with viable pollen (blue shadow) with reduced pistil development.

The second level distinguishes female and male flowers, addressing the dioecious nature of *Actinidia chinensis*. Recognising the gender distinction is crucial, as each gender has distinct functions. While female flowers develop into kiwifruit after pollination, which directly impacts yield, male flowers serve exclusively as pollen sources during the pollination phase. There are distinct morphological characteristics of the reproductive organs of flowers that allow for clear visual identification of the gender^16^. These distinct morphological features allow accurate gender classification during data collection and annotation. Figure 3 identifies the key morphological characteristics of the reproductive organs to differentiate the genders.

**Fig. 4:**
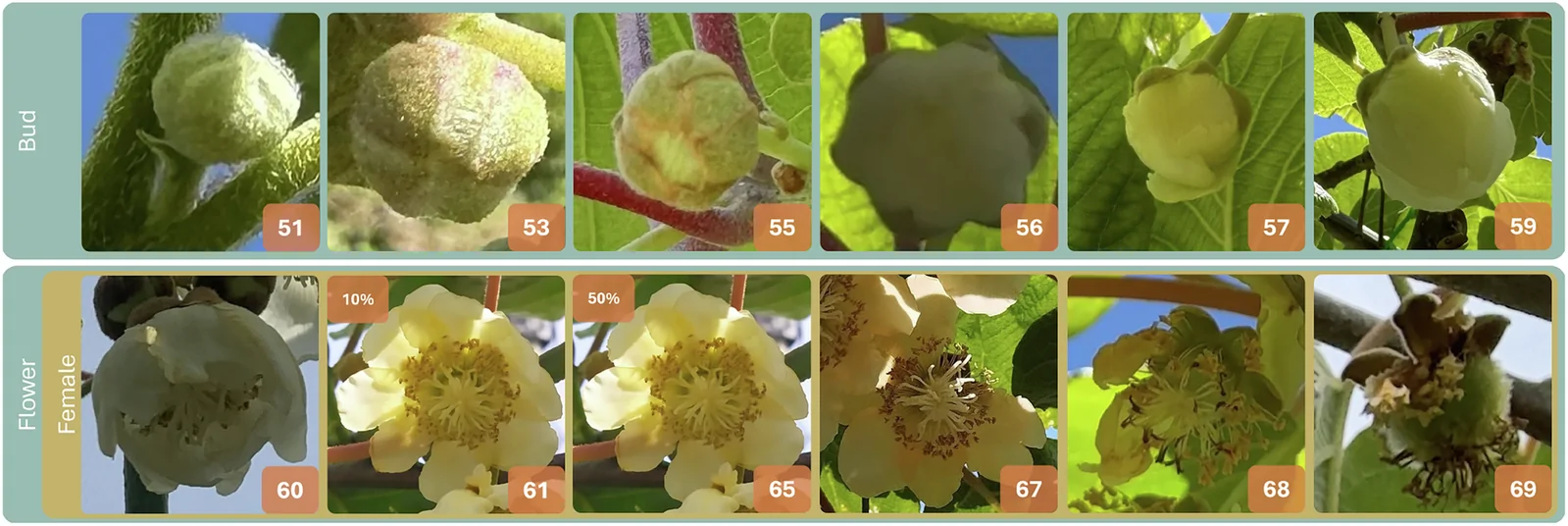
Examples of each phenological growth stage of *Actinidia chinensis* according to the BBCH scale from inflorescence bud swelling (stage 51) through fruit set (stage 69)^16^.

The third level is the most detailed classification level that implements standardised phenological staging according to the BBCH (Biologische Bundesanstalt, Bundessortenamt und CHemische Industrie) scale^17^ adapted for *Actinidia chinensis*^16^. The BBCH scale is characterised by a decimal-based framework that matches plant development into ten principal growth stages, which are subdivided into ten secondary stages (not all numbers need to correspond to a stage of development), enabling precise documentation of phenological events^18^. For this work, the principal growth stages of interest are the inflorescence emergence and flowering stages (stage 5 and stage 6, respectively)^16^. Figure 4 shows the twelve secondary phenological stages, using the defined colour-coded schema to delineate the hierarchical organisation, while Table 3 provides detailed descriptions of the key morphological characteristics for distinguishing the respective secondary stages.

**Table 3:** Criteria for distinguishing each phenological growth stage of *Actinidia chinensis* according to the BBCH scale from inflorescence bud swelling (stage 51) through fruit set (stage 69)^16^.

The BBCH scale has limitations for dioecious species. Male flowers are not included, as they fulfil their function by releasing pollen without developing fruit, creating gaps in the phenological classification. Furthermore, some stages (stage 61 and stage 65) describe flowering patterns at the level of the orchard’s flower population, rather than individual flower characteristics.

The Multi-Modal *Actinidia chinensis* Phenology Dataset classes were defined according to the BBCH scale, and some adaptations were made to resolve the mentioned limitations.

- In the case of buds, stage 51 was excluded since these small buds without a peduncle were frequently occluded by the foliage. Stage 57 and stage 59 were combined into class bud_57, as they had minimal morphological differences, making identification difficult.
- In the case of female flowers, stage 61 and stage 65 at the population level were adapted to individual flower characteristics, with flower_female_61 redefined as fully open flowers.
- In the case of male flowers, female flower stages were adapted to include male flower development stages (flower_male_60, flower_male_61, flower_male_67).

**Fig. 5:**
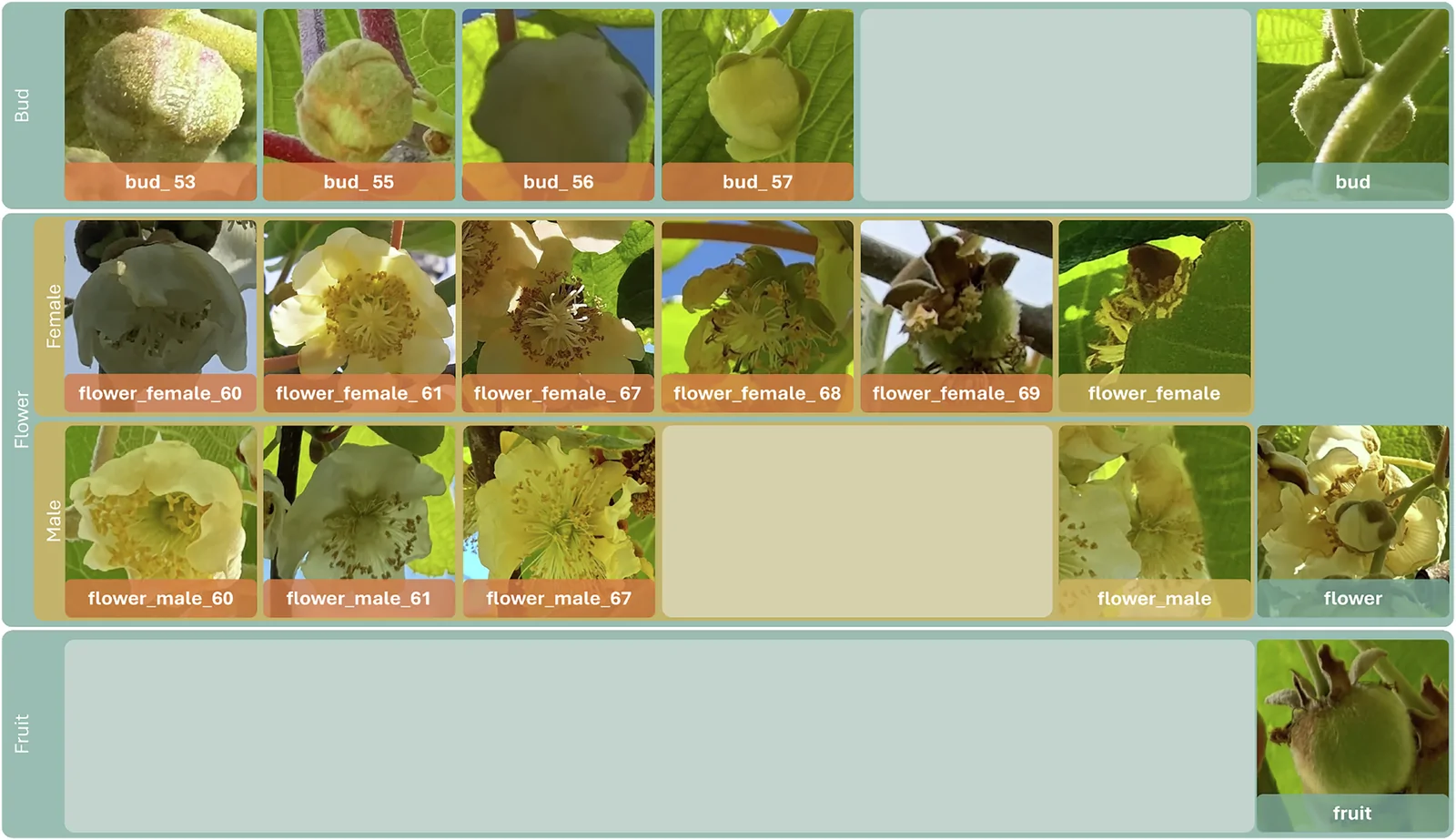
Examples of each class, including specific classes for each phenological stage (from swelling of inflorescence buds (bud_53) to fruiting (flower_female_69) and generic classes for gender (flower_female and flower_male) and structure (bud, flower and fruit).

The resulting system comprises 17 classes that combine specific BBCH stage categories with generic structural classes to accommodate cases where stage or gender determination is not feasible due to image quality, viewing angle, or developmental ambiguity. Figure 5 presents the 17 classes using the colour-coded hierarchical schema. Table 4 provides morphological criteria for each class and summarises the correspondence with standard BBCH stages.

**Table 4:** Criteria for distinguishing each class and correspondence to standard BBCH stages.

Male flower classes are dataset-specific extensions, and generic classes have no BBCH equivalent.

**Fig. 6:**
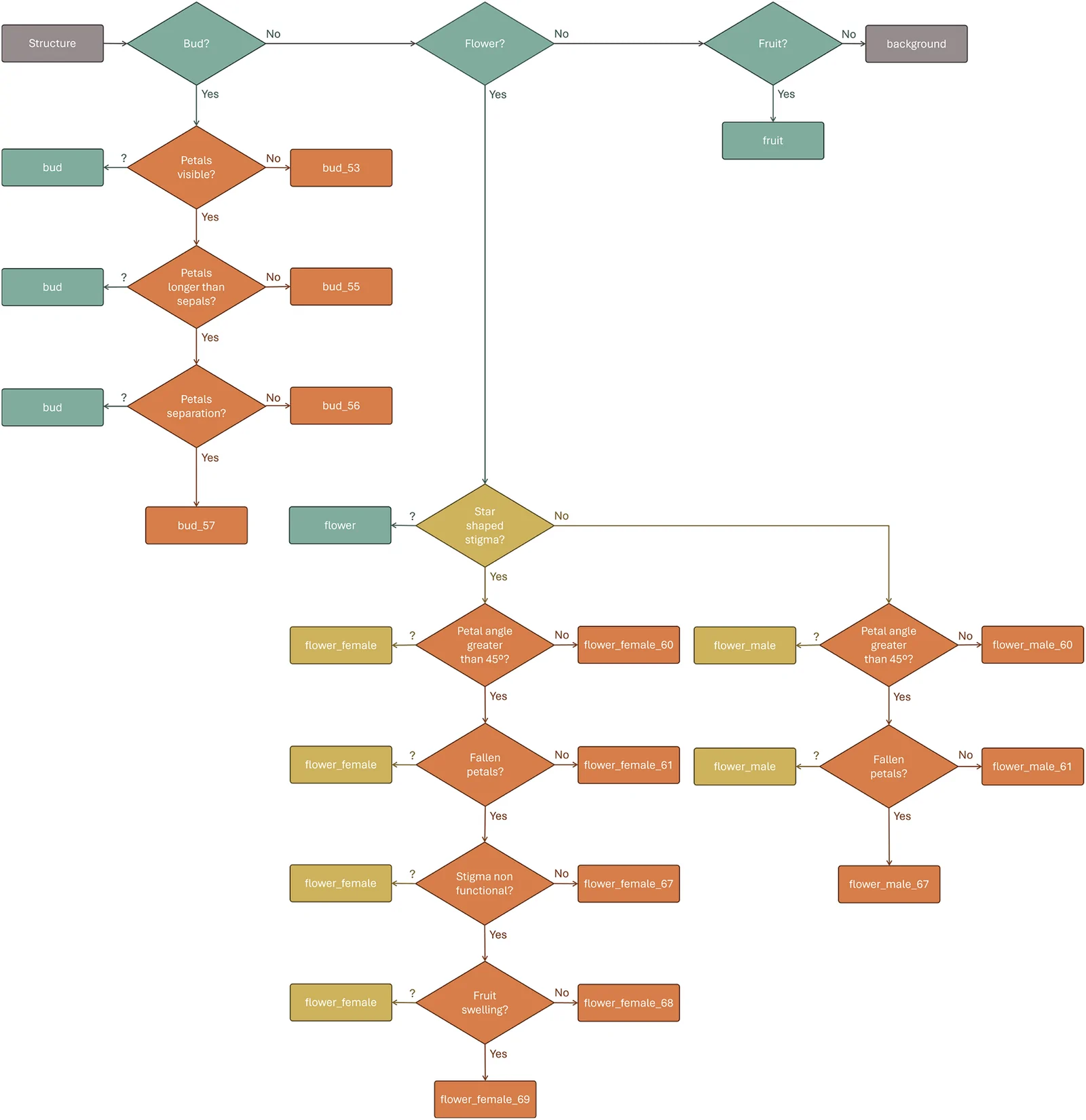
Annotation decision flowchart guiding classification from region of interest detection to final class assignment. Uncertain cases at any decision point are assigned to the appropriate generic class via “?” paths.

To ensure consistent classification across both dataset components, a structured annotation protocol was developed with explicit decision criteria for distinguishing between phenological stages. Figure 6 presents the complete decision flowchart guiding the classification process.

Starting from a structure, annotators first evaluate its type by sequentially assessing whether it corresponds to a bud, a flower, or a fruit. Structures that do not match any category are classified as background. The decision tree employed sequential morphological criteria specific to each structure type. For buds, annotators evaluated: (i) petal visibility through sepals, (ii) petal extension beyond sepal margins, and (iii) petal separation without visible stigma. For flowers, annotators first determined gender by identifying the star-shaped stigma characteristic of female flowers. For female flowers, annotators evaluated: (i) extent of petal opening, (ii) presence of petal fading, (iii) stigma functionality, and (iv) fruit development. Male flowers were assessed using the first two criteria, as they do not develop fruit. At each decision point, when annotators cannot confidently determine the answer, the structure is assigned to the appropriate generic class via the “?” paths, ensuring systematic handling of transitional or ambiguous instances.

The Multi-Modal *Actinidia chinensis* Phenology Dataset comprises two complementary components utilising these 17 classes. The first component consists of manually annotated images with bounding box labels for CV model training and validation. The second component includes georeferenced videos with systematic phenological stage counts across defined spatial areas, providing ground truth data for spatial distribution analysis and monitoring applications.

#### Labelled Images Component

Manual annotation was performed using the Computer Vision Annotation Tool (CVAT)^19^ following the decision flowchart presented in Fig. 6. Bounding boxes tightly enclosed each structure with minimal margins, excluding peduncles to focus on diagnostic morphology. Structures were annotated when at least 50% of defining characteristics were visible and unoccluded, permitting partial overlap between adjacent structures. The dataset includes images with single or multiple classes, as well as background images without target structures.

Annotation was performed by a primary annotator across all 1 665 images over three consecutive days to maintain temporal consistency. Independent validation was conducted by a trained secondary annotator on a randomly selected 10% subset (167 images), as detailed in Technical Validation.

Images were exported in Pascal Visual Object Classes (VOC) format, generating XML files with annotation metadata including bounding box coordinates, class assignments, and image dimensions. Table 5 presents the distribution of images and annotations across individual classes and hierarchical levels, with the colour scheme reflecting the hierarchical structure.

**Table 5:** Number of images and annotations for each class in the Multi-Modal Actinidia chinensis Phenology Dataset.

Colour coding reflects hierarchical levels: structure (blue), gender (yellow), and phenological stage (orange).

#### Georeferenced Videos Component

Manual phenological assessment followed the decision criteria established in Fig. 6. Unlike image-based annotation, field observers had direct access to structures from multiple viewing angles, enabling confident determination of both gender and phenological stage. Consequently, generic classes were unnecessary, and all structures were classified into the 13 stage-specific classes. For model validation workflows, users should evaluate stage-specific predictions against the 13-class ground truth and report generic class predictions separately as structures where a limited perspective prevented confident assignment.

Ground truth counting utilised a structured data matrix with individual plant shoots as rows and phenological stage classes as columns. Operators systematically inspected each sampling zone, cross-checking ambiguous structures from different angles and recording only clearly identifiable phenological features. Manual counts were digitised and compiled into standardised Excel files for each sampling zone, providing zone-level ground truth data corresponding to the collective set of six videos per zone.

Table 6 presents spatial distribution patterns and validation counts across sampling zones and phenological classes, with colour coding reflecting the hierarchical classification structure.

**Table 6:** Ground truth counts across sampling zones showing hierarchical aggregation levels for the georeferenced videos component.

Colour coding reflects hierarchical levels: structure (blue), gender (yellow), and phenological stage (orange).

## Data Record

**Fig. 7:**
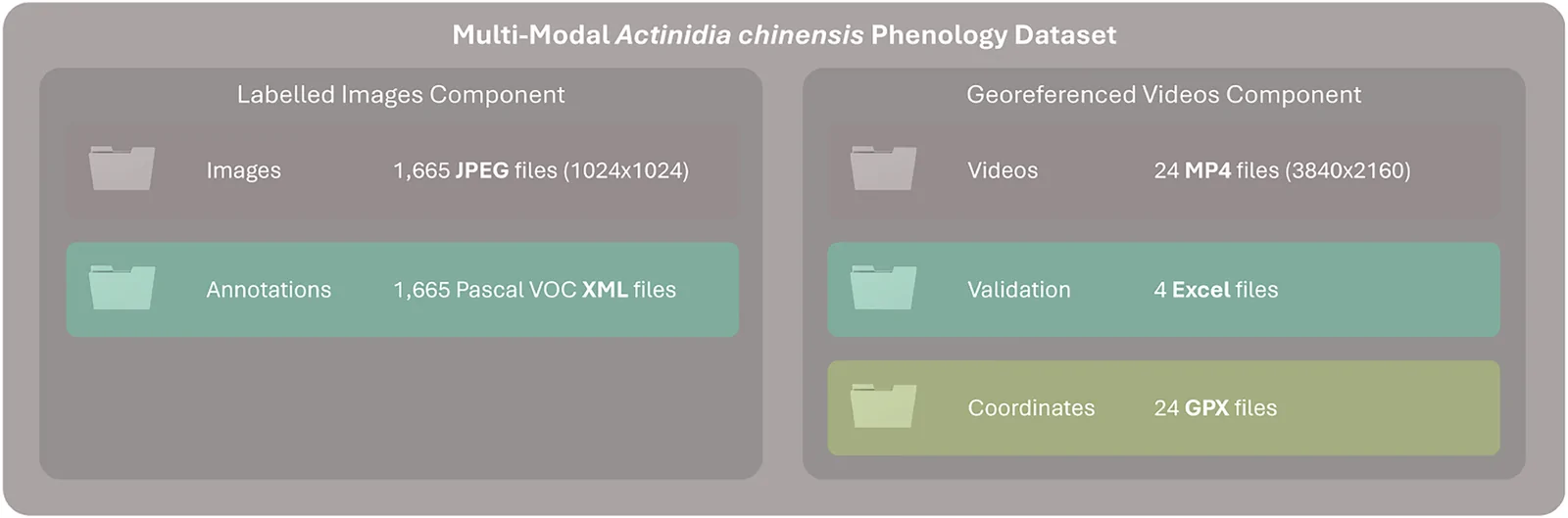
Multi-Modal *Actinidia chinensis* Phenology Dataset architecture showing file organisation and specifications for both components.

The Multi-Modal *Actinidia chinensis* Phenology Dataset^11^ is publicly available at Zenodo. The dataset comprises 1 665 annotated images and 24 georeferenced video segments, with corresponding ground truth validation data, structured to support hierarchical phenological classification and spatial mapping applications. Figure 7 illustrates the dataset architecture and file organisation.

The labelled images component contains JPEG files standardised to 1024 × 1024 pixels with corresponding Pascal VOC XML annotations. Each XML file contains bounding box coordinates (xmin, ymin, xmax, ymax) and class labels from 17 categories encompassing stage-specific BBCH classifications and generic morphological groups.

The georeferenced videos component comprises 24 MP4 files distributed across four sampling zones (A-D) with six videos per zone. Each video has a corresponding GPX file containing track points with WGS84 coordinates, timestamps at 1-second intervals, and metadata linking to the source video. Frame-level spatial referencing is achieved by linearly interpolating between the recorded start and end positions.

Manual validation data are provided as Excel files, one per sampling zone, with plant shoots as rows and 13 stage-specific BBCH categories as columns. Generic morphological classes are excluded as field protocols enabled assignment to specific developmental stages.

## Technical Validation

### Labelled Images Component

Annotation quality was assessed through a systematic validation protocol. A primary annotator labelled all 1 665 images over three consecutive days to maintain temporal consistency, following the classification criteria established in Table 4 and the decision flowchart in Fig. 6.

To assess inter-annotator reliability, a secondary annotator completed four hours of structured training and independently annotated a randomly selected 10% subset (167 images) without access to primary annotations. Inter-annotator agreement was quantified using Cohen’s Kappa coefficient (*κ* = 0.8) for class labels and mean Intersection over Union (*mIoU* = 0.7) for bounding box localisation^20^.

The 10% validation confirms protocol consistency but does not assess systematic annotator bias. Fine-grained distinctions between consecutive stages require subjective judgment, and threshold decisions reflect primary annotator interpretation. Additionally, the dataset exhibits class imbalance reflecting natural phenological occurrence, with female flower stages more represented than male and late senescence stages underrepresented (Table 5). Users should consider class-weighted approaches for model training and account for potential bias toward dominant classes when interpreting performance metrics.

### Georeferenced Videos Component

Manual phenological assessments were conducted in the field by operators following the classification criteria in Table 4 for 13 stage-specific classes. Generic classes were excluded as field conditions permitted detailed morphological assessment across multiple viewing angles, enabling reliable assignment to specific developmental stages. Manual counts are provided as Excel files containing plant-level phenological stage assignments for each sampling zone.

The spatial validation data represent a single orchard and serve as a methodological framework that users can replicate in additional orchards to extend spatial coverage. Class distribution is uneven, with male flowers absent from three of four sampling zones due to orchard spatial arrangement (Table 6). Users validating detection models against spatial counts should account for this uneven distribution across genders and stages.

## Data Availability

The Multi-Modal *Actinidia chinensis* Phenology Dataset described in this Data Descriptor is publicly available at Zenodo: https://doi.org/10.5281/zenodo.19683529. This dataset comprises two components: (1) 1 665 JPEG images (1024 × 1024 pixels) with corresponding Pascal VOC XML annotation files containing bounding box coordinates and phenological class labels, and (2) 24 MP4 video files (3840 × 2160 pixels) with corresponding GPX coordinate files and Excel validation files containing manual ground truth counts.

## Code availability

Custom scripts for dataset preparation are publicly available under the MIT License at https://github.com/INESCTEC/actinidia-dataset-preprocessing. These scripts implement standard operations, including image resolution standardisation to 1024 × 1024 pixels (reproducible from original raw images) and GPX track generation for video georeferencing through linear interpolation (reproducible from original videos and coordinate logs). The scripts are provided for transparency and to enable users to apply similar preprocessing to their own data.

Image annotation was conducted using CVAT (https://github.com/opencv/cvat). Uniqueness calculations for duplicate detection were performed using the Fiftyone platform (https://github.com/voxel51/fiftyone).
